# SEMA6D, Negatively Regulated by miR-7, Contributes to C28/I2 chondrocyte's Catabolic and Anabolic Activities via p38 Signaling Pathway

**DOI:** 10.1155/2022/9674221

**Published:** 2022-06-16

**Authors:** Haoyu Yang, Zhicheng Yang, Zhentang Yu, Chenwei Xiong, Yi Zhang, Junjie Zhang, Yong Huang, Xindie Zhou, Jin Li, Nanwei Xu

**Affiliations:** ^1^Department of Orthopedics, Wuxi 9th People's Hospital Affiliated to Soochow University, Wuxi 214000, China; ^2^Department of Orthopedics, The Affiliated Changzhou No.2 People's Hospital of Nanjing Medical University, Changzhou 213000, China; ^3^Department of Orthopedic Surgery, The Second Affiliated Hospital of Jiaxing University, Jiaxing 314000, China

## Abstract

MiR-7 has been recognized as an osteoarthritis (OA-)-promoting factor, but the specific downstream pathway of miR-7 still remains unknown. Further investigation of the molecular regulatory mechanism of miR-7 might help develop a novel therapeutic method for OA. In this study, we revealed that Semaphorin 6D (SEMA6D) was a direct target gene of miR-7 and presented a negative regulatory relation with SEMA6D *in vitro* and *in vivo*. SEMA6D could improve OA in rat OA models, as indicated by H&E and Safranin O-Fast green staining, and also *μ*CT analysis. Further evaluation of SEMA6D suggested that SEMA6D promotes the anabolism and reduces the catabolism of C28/I2 chondrocytes via inhibiting the activation of the p38 pathway. The present research illustrated that SEMA6D is a negatively regulatory factor of miR-7 and a pivotal mediator of catabolism and anabolism in C28/I2 chondrocytes. SEMA6D exerts its function via inhibiting the activation of the p38 pathway.

## 1. Introduction

Osteoarthritis (OA) has been recognized as a degenerative chronic disease with high incidence that mostly occurs in senior citizens and is characterized by cartilage degeneration and alteration of secondary subchondral bone, thereby inducing articulus malfunction [1–3]. OA has now become a predominant reason for a low standard of living, which is usually manifested as pain and functional limitations caused by the degeneration of articular cartilage [4]. According to epidemiological research data, the incidence of OA in the elderly aged over 55 years was higher than 50%, and a much higher incidence rate (over 60%) was observed in individuals aged over 65 years [5]. The complexity of the etiology and pathogenesis of OA is correlated with many pathological factors including heredity, metabolism, trauma, and old age, among which old age is considered to be the most important one [6, 7]. With the rapid development of aging population, there is an urgent need for OA treatment strategies to improve the quality of life of the population. Therefore, searching for effective therapeutic strategies for treating OA has been a research hotspot.

Previous studies have revealed that the imbalance of anabolic and catabolic activities of C28/I2 chondrocytes is a predominant factor in OA. Accordingly, further investigation of the cytoskeletal integrity and factors influencing anabolic and catabolic activities is being conducted to reveal the molecular mechanism of OA [8]. Recently, accumulating evidence has proved that microRNAs (miRNAs) participate in regulating the development of OA [9]. miRNA is a type of noncoding RNA capable of mediating cell differentiation, apoptosis, and tumorigenesis [10]. Previous studies have discovered that miRNA also serves a functional role in mediating the metabolism of cartilage matrix and chondrocytes. Research reports have elucidated to date that miRNA regulatory network, chondrocytes autophagy, and alteration in epigenetics would be the targeted therapeutic strategies for OA [11]. In addition, our previous research discovered that miR-7 was overexpressed in IL-1*β*-induced osteoarthritic C28/I2 chondrocytes by activating PI3K/AKT/mTOR and thereby worsened the course the OA [12]. However, the specific molecular mechanisms of miR-7 still remain unknown, causing a hindrance in developing a novel treatment method of osteoarthritis.

Semaphorin 6D (SEMA6D) is a member of the semaphorin family, which serves as a crucial factor in mediating nervous system development [13, 14]. Nowadays, SEMA6D has been recognized as regulatory factor in multiple physiological processes and the development of diseases, including the development of visual system, maturation of perinatal cardiomyocytes, and development of invasive breast carcinoma [15, 16].

However, so far, there is little data concerning the exact function of SEMA6D in OA. In this study, we adopted multiple databases for the bioinformatics prediction of the downstream target gene of miR-7 and found that SEMA6D may be a potential target of miR-7. Further verification of this hypothesis was performed using the dual luciferase assay. We proposed an assumption that miR-7 promotes chondrocyte anabolic activity by mediating SEMA6D. Our findings indicate that SEMA6D is negatively regulated by miR-7 and thereby contributes to a balance between catabolic and anabolic activities via specific downstream pathways. These discoveries may provide novel therapeutic methods for OA patients.

## 2. Methods and Materials

### 2.1. Cell Culture

Normal human chondrocyte cell line C28/I2 was incubated with 90% high-glucose DMEM (HyClone, USA) with 10% FBS (Gibco, USA) at 37°C with 5% CO_2_. HY-12839, a p38 MAPK inhibitor, was purchased from MedChemExpress (NJ, USA).

### 2.2. Dual Luciferase Assay

To construct the reporter plasmids of wild-type (WT) and mutated (Mut) SEMA6D, pGL3 vector (Promega Corporation, Madison, USA) and synthetic SEMA6D with wild-type (WT) or mutated (Mut) regions were adopted. Subsequently, the reporter plasmids and miR-7 mimics were co-transfected into C28/I2 chondrocytes using Lipofectamine 2000. MiR-NC was used as a negative control. The dual-luciferase reporter gene assay system was then used to estimate the Renilla and Firefly luciferase activities after 24 h.

### 2.3. Cell Transfections

To construct C28/I2 chondrocytes with different expression quantities of SEMA6D, small interfering RNA-SEMA6D and pcDNA3.1-SEMA6D (Genechem, Shanghai, China) were synthesized and transfected into C28/I2 chondrocytes using Lipofectamine 2000 (Thermo Fisher Scientific), according to the manufacturer's protocol, to induce the silencing and up-regulation of SEMA6D, respectively. Lip2000-NC was used as a negative control.

### 2.4. Quantitative Reverse-Transcription PCR (qRT-PCR)

TRIzol Reagent was used for the extraction of total RNA from C28/I2 chondrocytes. Chloroform incubation was conducted for 15 min at room temperature and isopropyl alcohol was then added. Centrifugation was performed, and RNA was obtained from the precipitate. The expressions of miR-7, SEMA6D, Aggrecan, COL2A1, ID1, and Smad1 were analyzed using the PrimeScript RT reagent kit and SYBR Prime Script RT-PCR kits, according to manufacturer's instructions. The results were analyzed through 2^−*ΔΔ*ct^. GAPDH was used as the internal control. The primer sequences were as follows: hAggrecan, forward: 5′-GACTTCCGCTGGTCAGATGG-3′, reverse: 5′-CGTTTGTAGGTGGTGGCTGTG-3′; ID1, forward: 5′−GTGCCTAAGGAGCCTGGAAAA-3′, reverse: 5′-TTCAGCGACACAAGATGCGA-3′; MMP13, forward: 5′-CATGAGTTCGGCCACTCCTT-3′, reverse: 5′-CCTGGACCATAGAGAGACTGGA-3′; miR-7, forward: 5′−TGGAAGACTAGTGATTTTGTT-3′, reverse: 5′-CCAGTCTCAGGGTCCGAGGTATTC-3′.

### 2.5. Animal Experiments

The destabilized medial meniscus (DMM) model was employed to mimic OA. A total of 30 four-week-old male Sprague Dawley rats (200–250 g), the medial meniscuses of which were carefully resected without cartilage and ligament injuries, were randomly divided into different groups: NC group (no surgery; normal saline treatment, injection time same as the OA group; 10 knee joints from 5 rats, *n* = 10); OA group (surgery; normal saline treatment on the first day of every week from the 5th to 8th week after surgery); OA+lip2000-NC group (surgery; 100 *μ*L normal saline with 1 × 10^9^ PFU lip2000-NC, injection time same as the OA group; 5 rats, *n* = 10); OA+ small interfering RNA SEMA6D (si-SEMA6D) group (surgery; 100 *μ*L normal saline with 1 × 10^9^ PFU lip2000-NC of SEMA6D, injection time same as the OA group; 5 rats, *n* = 10); OA+pcDNA3.1 group (surgery; 100 *μ*L normal saline with 1 × 10^9^ PFU pcDNA3.1, injection time same as the OA group; 5 rats, *n* = 10); and OA+pcDNA3.1-SEMA6D group (surgery; 100 *μ*L normal saline with 1 × 10^9^ PFU pcDNA3.1 of SEMA6D, injection time same as the OA group; 5 rats, *n* = 10). After treatment, rats were sacrificed by an overdose of anesthesia, and knee samples were obtained and fixed with 4% paraformaldehyde for at least 48 h. The study was performed according to NIH guidelines (NIH Pub No 85-23, revised 1996) and the Laboratory Animal Management Regulations in China and adhered to the Guide for the Care and Use of Laboratory Animals published by the National Institutes of Health (2011). The study protocol was approved by the Ethics Committee of the Affiliated Changzhou No.2 People's Hospital of Nanjing Medical University (Changzhou, China), and written informed consent was obtained from all participating patients.

### 2.6. Human Tissue Sample Collection

OA articular cartilage samples and healthy cartilage samples from trauma patients without OA were collected from the Department of Orthopedics of the Affiliated Changzhou No.2 People's Hospital of Nanjing Medical University (Changzhou, China).

### 2.7. Micro-CT Analysis

SD rats were euthanized, and their knee joint specimens were collected and fixed with 10% formalin. The specimens were analyzed via micro-CT with the following parameters: 18 *μ*m, 65 kV, and 385 mA. Further, 3D reconstruction of the scanned knee joint images was performed using the Mimic software. Appropriate 3 mm cylindrical regions were designated as regions of interest (ROI) using Dataview and CT analyzer softwares.

### 2.8. Histological (H&E and Safranin O-Fast Green Staining) and Immunohistochemical Analyses

Knee joint tissues were fixed in 4% paraformaldehyde for 48 h, decalcified with 10% formic acid (commercially available decalcification solution) for 10 days, dehydrated with graded ethanol, and embedded in paraffin. Serial mid-sagittal sections (3 *μ*m thick) were cut and stained with H&E and Safranin O-Fast green for morphological analysis. Fixation, dehydration, paraffin embedding, and sectioning were performed according to the standard of pathological examination, followed by dewaxing, dehydration, staining, and sealing. The dried sections were treated under an automatic immunohistochemical staining machine and then sealed with neutral gum. The test was completed according to the instructions mentioned in the kit. The sections were photographed and observed using a Canon microscopic imaging system (model EOS-350D, Canon, Tokyo, Japan).

### 2.9. Induction of Active Catabolism and Anabolism in C28/I2 Chondrocytes

For evaluating the specific role of SEMA6D in C28/I2 chondrocytes, fibronzetin fragment (FN-f) and osteopenia protein-1 (OP1) were used to induce catabolism and anabolism in C28/I2 chondrocytes, respectively. FN-f with different concentrations (0, 0.5, 1.0, and 2.0 *μ*M) was used to determine an optimal concentration. Further, 0, 50, 100, and 150 ng/ml of OP1 were used to find a favorable concentration.

### 2.10. Western Blot Assay

Cells in the exponential growth period were collected and washed with PBS three times. Cells were then lysed using a lysis buffer (Beyotime, Nanjing, China) on ice for 30 min. The BCA kit (Beyotime, Nanjing, China) was used to calculate the total protein concentration. Loading buffer (Beyotime, Nanjing, China) was added to the protein solution and boiled for 5 min. After separation via SDS-PAGE, proteins were transferred onto polyvinyl difluoride membranes (EMD Millipore, Billarica, MA, USA) and were then incubated with primary antibodies (including SEMA6D, MMP-2, Smad1, p-Smad1, ERK, p-ERK, p38, p-p38, Aggrecan, COL2A1, and ID1) and subsequently secondary antibodies. GAPDH was adopted as the negative control. Finally, to observe the intensity of proteins' bands, an enhanced chemiluminescence reagent (Thermo Scientific, Waltham, MA, USA) was used. The grey intensity was analyzed via image J and GraphPad prism software.

### 2.11. Immunofluorescence Examination

To further estimate the expression quantities of SEMA6D in different groups of C28/I2 chondrocytes, cells were cultured to reach a confluence of roughly 80% after transfection. PBS was used to wash cells three times, and the cells were then fixed using cold methanol for 20 min. Subsequently, 0.1% Triton X-100 was used to permeate the fixed cells. The primary antibody of COL2A1 was then used to treat cells at 4°C for 24 h. A secondary Alexa Fluor® 594-conjugated antibody was added and maintained at 25°C for another 1 h. Following this, DAPI was used to stain the nucleus for 5 min. Finally, the fluorescence intensity was estimated using the Leica fluorescence microscope.

### 2.12. Statistical Analyses

The experimental data were statistically analyzed using SPSS 20.0 (IBM Corp., Armonk, NY, USA) and GraphPad 7 (GraphPad Software, San Diego, CA, USA). Student *t* test was used to compare between-group statistical differences, and multiple groups were compared using one-way analysis of variance. *p* < 0.05 means the significance of statics.

## 3. Results

### 3.1. SEMA6D Is a Downstream Target Gene of miR-7 in C28/I2 Cells

**Figure 1:**
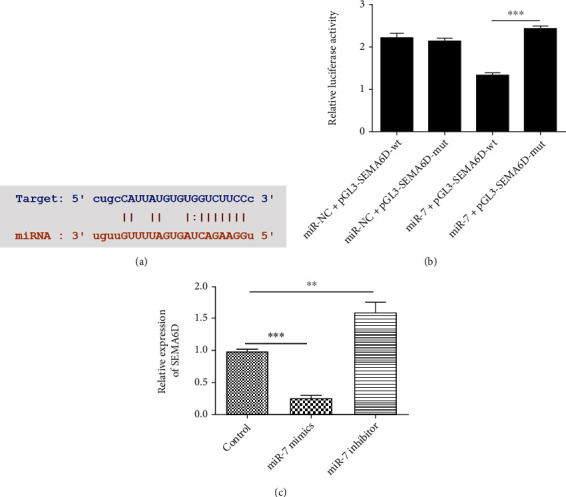
SEMA6D was recognized as a negative regulatory target of miR-7. (a) Bioinformatics prediction showed an overlapping sequence between miR-7 and SEMA6D. (b) The results of dual-luciferase assay demonstrated the existence of a direct interaction between miR-7 and SEMA6D. (c) qRT-PCR indicated that SEMA6D level was down-regulated when there was an overexpression of miR-7, and SEMA6D level increased when miR-7 was down-regulated. ∗∗ indicates *p* < 0.01 between groups. ∗∗∗ indicates *p* < 0.001 between groups.

Our previous studies have already proved that miR-7 is down-regulated in normal chondrocytes and thereby exacerbates the course of OA through aggravating cartilage degradation [12]. Nonetheless, the downstream regulatory target gene still remains unknown. Further investigation of the regulatory axis of miR-7 could possibly help in developing novel therapeutic methods for OA. This encouraged us to continue research on miR-7. Multiple databases including TargetScan and starBase were adopted to determine the target gene of miR-7, and SEMA6D was found to be a potential regulatory protein of miR-7. Bioinformatics prediction was conducted to discover that there was a direct binding site for miR-7 on SEMA6D sequence (Figure 1(a)), suggesting the possibility of the existence of interaction between SEMA6D and miR-7. To further confirm this assumption, dual luciferase assay was performed, and a significant difference between SEMA6D-Mut group and SEMA6D-WT group was observed, wherein both were co-treated with miR-7 mimic (Figure 1(b), *p* < 0.01). This result verified that there was a direct interaction between miR-7 and SEMA6D. To identify the exact relation be miR-7 and SEMA6D, miR-7 mimics and miR-7 inhibitor were administrated to C28/I2 chondrocytes. Subsequently, the relative transcription level of SEMA6D was evaluated through qRT-PCR, and the result demonstrated that up-regulation of miR-7 led to suppression of SEMA6D transcription in OA chondrocytes, whereas the miR-7 inhibitor group presented the opposite phenomenon (Figure 1(c), *p* < 0.001). This suggested that SEMA6D was negatively regulated by miR-7 in chondrocytes.

### 3.2. Mir-7 Accelerated the Development of OA In Vivo by Down-Regulating SEMA6D

**Figure 2:**
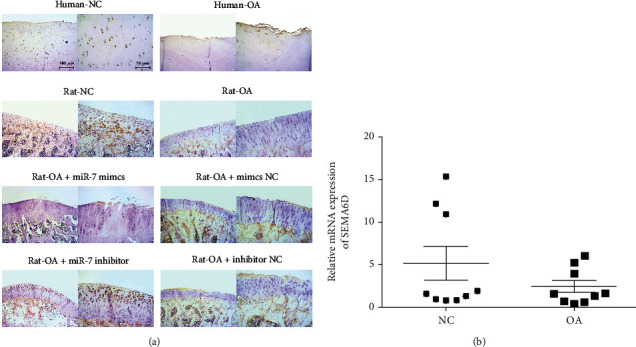
SEMA6D was negatively regulated by miR-7 *in vivo*. (a) The immunohistochemical analysis results of SEMA6D in human and rat cartilage tissues in different groups. (b) qRT-PCR indicated that SEMA6D was up-regulated in normal cartilage tissues compared with OA tissues; ∗*p* < 0.05.

Further understanding the specific role of SEMA6D *in vivo*, we evaluated human cartilage tissues and rat cartilage tissues.  Figure 2(a) shows the immunohistochemical consequences of SEMA6D in human and rat cartilage tissues; the results showed that SEMA6D expression level was higher in both normal human and rat cartilage tissues compared with that in OA cartilage tissues. Next, to further verify the correlation of miR-7 with SEMA6D *in vivo*, miR-7 mimics and miR-7 inhibitor were administered to normal rats and OA rats to investigate SEMA6D expression, and negative controls (mimics-NC and inhibitor-NC) were also administered to OA rats. Research results showed that the up-regulation of miR-7 decreased the expression levels of SEMA6D *in vivo* when compared with the mimic-NC group, whereas the administration of miR-7 inhibitor in OA rats resulted in lower SEMA6D levels, confirming the negative regulatory effect of miR-7 on SEMA6D. The statistical analysis of SEMA6D expression in normal and OA rat tissues is shown in Figure 2(b), which conspicuously indicated the lower-than-usual expression levels of SEMA6D in OA chondrocytes. These results possibly suggested that SEMA6D may have pathological regulatory function in the course of OA.

### 3.3. SEMA6D Improved OA in Rat OA Models

**Figure 3:**
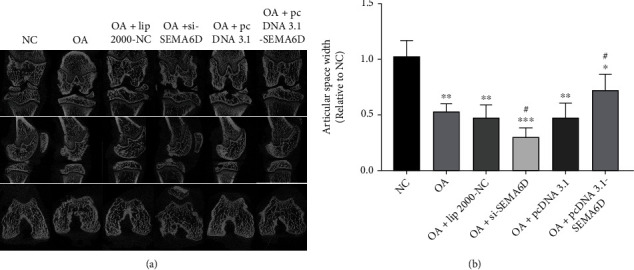
SEMA6D reduces the joint space narrowing *in vivo*. (a) Representative micro-CT images of knee joints in six groups of rats after DMM surgery with different treatments. (b) The relative articular space width in different groups, as determined from the micro-CT images. (*n* = 5, ∗ and ^#^ represent *p* < 0.05 on comparing with NC and OA groups, respectively).

To further validate the effect on SEMA6D *in vivo*, a DMM rat OA model was developed to evaluate the corresponding effects *in vivo*. Different plasmids were injected into the knee joints of OA rats. The representative CT radiographs of rat knee joints after treatments (Figure 3(a)) indicated the morphology of all rat knee joints. The articular space widths in the five OA groups (Figure 3(b)) became narrower after surgery compared with those in the NC group. This result indicates the successful establishment of the OA model by the DMM surgery. Importantly, the articular space width in the pcDNA3.1-SEMA6D group was much higher than that in the OA group, whereas si-SEMA6D shortened the space, suggesting that SEMA6D is beneficial for inhibiting OA development.

**Figure 4:**
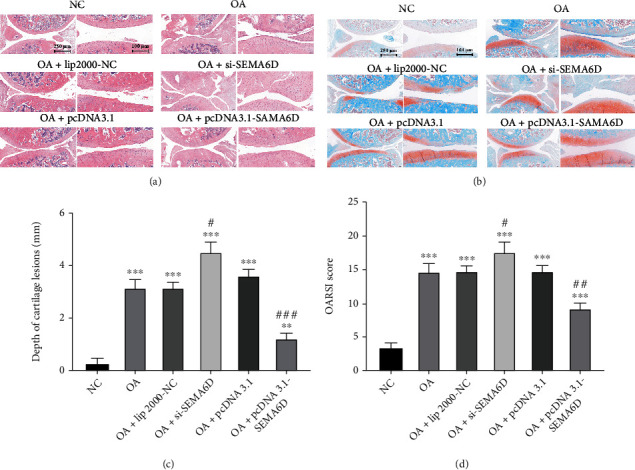
SEMA6D delayed the progression of osteoarthritis *in vivo*. Representative images of H&E staining (a). and Safranin O-fast green staining (b). in different groups. (c) The corresponding depth of cartilage lesions, and (d) OARSI score of articular cartilage in different groups of rats (*n* = 5, ∗ and ^#^ represent *p* < 0.05 on comparing with NC and OA groups, respectively).

The cartilage was collected for histological evaluation. The results of H&E staining (Figure 4(a)) and Safranin O-Fast green staining (Figure 4(b)) indicated severe cartilage destruction in the OA group. Surgical resection-induced cartilage destruction in the pcDNA3.1-SEMA6D group was remarkably alleviated compared with that in the OA group, whereas the destruction in the OA+si-SEMA6D group was significantly worsened. It was noted that the depth of cartilage lesions (Figure 4(c)) and OARSI score (Figure 4(d)) in the pcDNA3.1-SEMA6D group greatly decreased compared with those in the OA group, whereas these factors were elevated in the OA+si-SEMA6D group, indicating that SEMA6D can effectively prevent the deterioration of cartilage tear.

### 3.4. SEMA6D Was Overexpressed in C28/I2 Cells with Strong Anabolism

**Figure 5:**
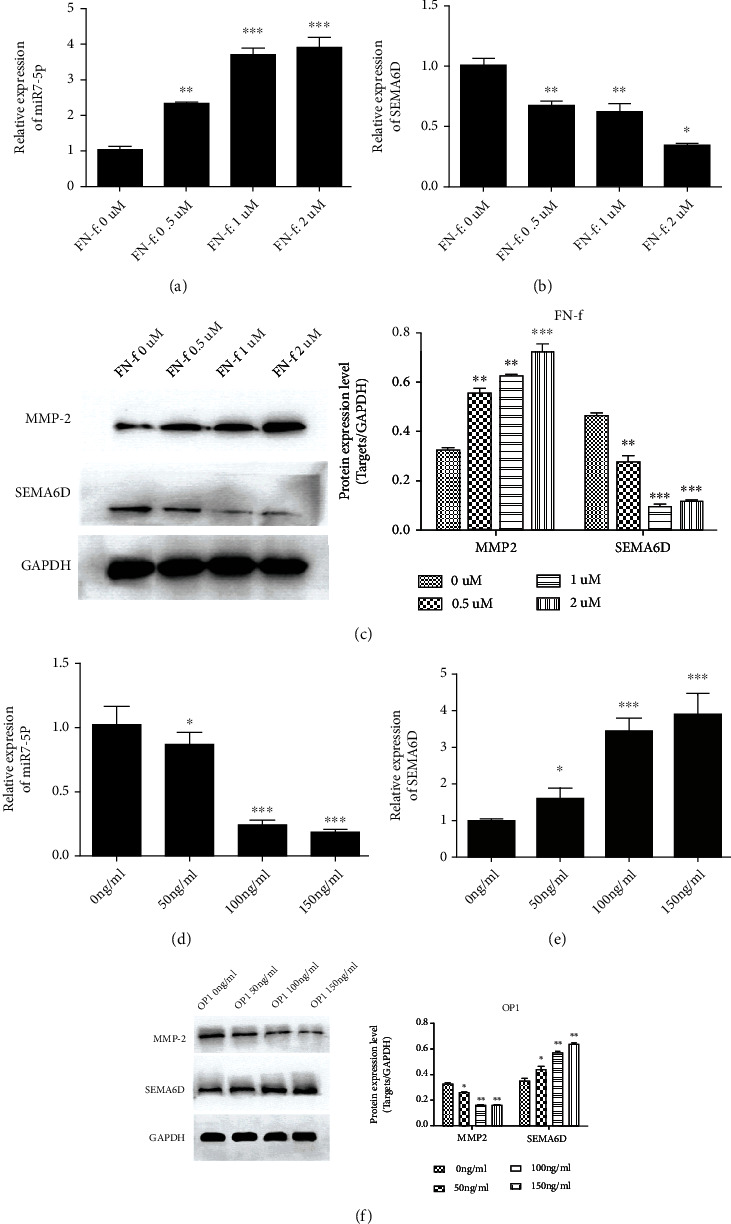
Investigation of SEMA6D in chondrocytes with active catabolism or anabolism. (a–b) Chondrocytes were stimulated with different concentrations of FN-f, and the quantities of miR-7 and SEMA6D were measured by qRT-PCR. (c) The expression levels of MMP-2 and SEMA6D were estimated via western blotting after treating with different concentrations of FN-f. (d–e) Chondrocytes were stimulated with different concentrations of OP1, and the levels of miR-7 and SEMA6D were measured by qRT-PCR. (f) The expression levels of MMP-2 and SEMA6D were estimated via western blotting after treatment with different concentrations of OP1. ∗∗∗*p* < 0.001, ∗∗*p* < 0.01, ∗*p* < 0.05 compared with control group.

The development of OA has been reported to be closely correlated with the anabolic and catabolic activity of C28/I2 chondrocytes [17, 18]. Multiple inflammatory mediators and mechanical stimulation collectively affect the physiological process of C28/I2 chondrocytes, thereby inducing excessive catabolic activity and triggering osteoarthritis [19–21]. Although previous experiments have demonstrated that miR-7 negatively regulates SEMA6D in OA chondrocytes, there is still much confusion about the roles of miR-7 and SEMA6D in mediating the development of OA. We next proposed a hypothesis that SEMA6D participates in regulating the catabolism and anabolism of C28/I2 chondrocytes. To confirm our assumption, we constructed C28/I2 chondrocytes with excessive catabolic activity using fibronectin fragment (FN-f) [22] and C28/I2 chondrocytes with excessive anabolic activity using osteogenic protein-1 (OP1) [8]. C28/I2 chondrocytes were treated with different concentration of FN-f (0, 0.5, 1, and 2 *μ*M) and OP1 (0, 50, 100, and 150 ng/ml) to induce catabolism and anabolism, respectively. Catabolism and anabolism induction by FN-1 and OP1, respectively, showed a dose-dependent trend. Subsequently, the transcriptional levels of miR-7 and SEMA6D were detected by qRT-PCR, and the results demonstrated that miR-7 was expressed at a highest level in the C28/I2 chondrocytes with highest catabolic activity level induced by FN-f, indicating that miR-7 possibly partakes in promoting the catabolism of OA chondrocytes (Figures 5(a) and 5(b)). In contrast, the expression levels of SEMA6D were the lowest in C28/I2 chondrocytes with highest catabolic activity. These results suggested that SEMA6D plays a role in preventing catabolism in C28/I2 chondrocytes, and the research results further illustrated that the regulatory effect of miR-7 and SEMA6D was opposite in C28/I2 chondrocytes. Subsequently, metalloproteinase 2 (MMP-2) was chosen as a biomarker in this study to examine the injury degree in cartilage cells with catabolism stimulation induced by FN-f as MMP-2 was shown to be up-regulated in OA chondrocytes with high level of catabolism [23]. The results of western blotting showed that MMP-2 was significantly up-regulated in the group treated with 2 *μ*M FN (Figure 5(c), *p* < 0.001), and SEMA6D was in the lowest expression level in that group (Figure 5(c), *p* < 0.001). These results showed that SEMA6D was down-regulated in C28/I2 chondrocytes with active catabolism, and SEMA6D may have a pathological correlation with the catabolic activity of OA chondrocytes. From the abovementioned research result, we could conclude that SEMA6D possibly participated in decreasing the catabolism of OA chondrocytes; however, whether SEMA6D is involved in promoting the anabolism of C28/I2 chondrocytes still remained unknown. Therefore, we used OP1 to induce active anabolism in C28/I2 chondrocytes and detected the transcriptional level of miR-7 and SEMA6D through qRT-PCR. The results demonstrated that 150 ng/ml of OP1 treatment presented the lowest level of miR-7, whereas the transcriptional quantities of SEMA6D were the highest (Figures 5(d) and 5(e), *p* < 0.001). Similarly, western blotting results showed that MMP-2 was down-regulated on OP1 treatment in a dose-dependent manner. In contrast, SEMA6D was significantly up-regulated upon OP1 treatment in a dose-dependent manner (Figure 5(f), *p* < 0.001). These results collectively illustrated that SEMA6D was pathologically correlated with catabolism and anabolism in C28/I2 chondrocytes and may act as a favorable factor in activating anabolism.

### 3.5. SEMA6D Reduced the Catabolism of OA C28/I2 Cells

**Figure 6:**
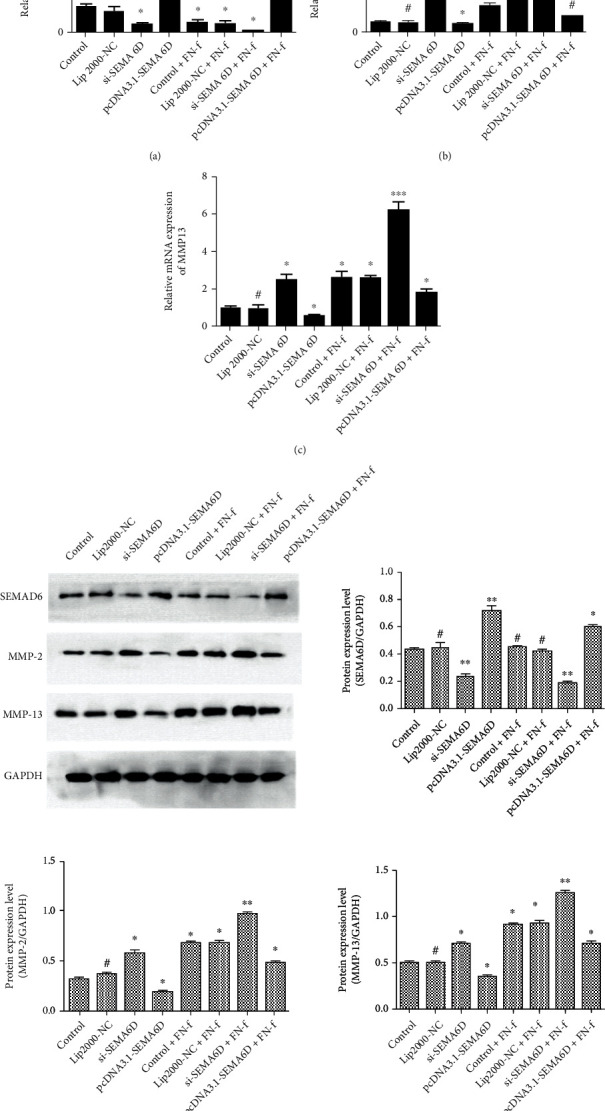
SEMA6D reversed the catabolism induced by FN-f. (a) The transfection effect of si-SEMA6D and pcDNA3.1-SEMA6D was verified by qRT-PCR. (b–c) Down-regulation of SEMA6D resulted in an overexpression of MMP-2 and MMP-13 both in normal chondrocytes and OP1-treated chondrocytes. (d) Western blotting was performed to measure the expression of MMP-2, MMP-13, and SEMA6D. ∗∗∗*p* < 0.001, ∗∗*p* < 0.01, ∗*p* < 0.05, and ^#^ indicates no significant difference compared with the control group.

According to literature research, the expression quantities of matrix metalloproteinases (MMPs), including MMP-2 and MMP-13, are increased in the cartilage tissues of OA patients [24, 25]. To further investigate the functional role of SEMA6D in OA chondrocytes, we constructed and transfected si-SEMA6D and pcDNA3.1-SEMA6D into chondrocytes for SEMA6D knockdown and overexpression, respectively. FN-f was used as an inducer to construct OA chondrocytes. Next, we examined the mRNA and protein expression levels of MMP-2, MMP-13, and SEMA6D via qRT-PCR and western blotting. After transfection of si-SEMA6D and pcDNA3.1-SEMA6D, we first evaluated the expression level of SEMA6D. The level of SEMA6D was significantly lower in si-SEMA6D transfected group than that in normal chondrocytes (Figure 6(a), *p* < 0.05), whereas pcDNA3.1-SEMA6D transfected C28/I2 chondrocytes showed significantly higher SEMA6D expression levels (Figure 6(a), *p* < 0.001). Next, the levels of SEMA6D in FN-f-induced OA chondrocytes were also detected via qRT-PCR. The results showed that the transcriptional level of SEMA6D in FN-f-induced OA chondrocytes (control+FN-f) was significantly lower than that in normal chondrocytes (control) (Figure 6(a), *p* < 0.05). Furthermore, OA chondrocytes transfected with si-SEMA6D by FN-f and pcDNA3.1-SEMA6D presented conspicuously lower and higher levels of SEMA6D, respectively. To evaluate the functional role of SEMA6D in C28/I2 chondrocytes with active catabolism, we then examined the level of MMP-2 and MMP-13. As shown in Figures 4(b) and 4(c), the knockdown of SEMA6D significantly increases the transcriptional level of MMP-2 and MMP-13 in normal chondrocytes, whereas up-regulation of SEMA6D via pcDNA3.1-SEMA6D transfection reduces the levels of MMP-2 and MMP-13 (*p* < 0.05). In contrast, in FN-f-induced OA chondrocytes (control+FN-f), the transcriptional quantities of MMP-2 and MMP-13 significantly increased (*p* < 0.05), indicating that FN-f successfully constructed OA chondrocytes. When si-SEMA6D transfection was performed to silence SEMA6D in OA chondrocytes, the levels of MMP-2 and MMP-13 were found to be the highest (Figures 6(b) and 6(c), *p* < 0.001), whereas up-regulation of SEMA6D using pcDNA3.1-SEMA6D resulted in decreased MMP-2 and MMP-13 levels. Subsequently, western blotting was performed to further confirm the suppressive effect of SEMA6D on MMP-2 and MMP-13. The results again showed that silencing of SEMA6D by si-SEMA6D both in normal and OA chondrocytes led to an increase in MMP-2 and MMP-13 levels (*p* < 0.01), whereas up-regulation of SEMA6D showed the opposite effect (Figure 6(d), *p* < 0.05). These results gave a joint clarification that SEMA6D may be a functional factor in preventing further damage to OA cartilage.

### 3.6. SEMA6D Promoted Anabolic Activity via Inhibiting the Activation of p38 Pathway

Although we have discovered that SEMA6D may serve as a favorable factor in preventing catabolism and accelerating anabolism in C28/I2 chondrocytes, the specific regulatory mechanism of SEMA6D was still unclear. Based on a previous literature study, p38 kinase is associated with the exacerbation of OA by accelerating the synthesis of inflammatory factors and MMPs [26, 27] and mediating C28/I2 chondrocyte apoptosis [28]. Similarly, overactivation of extracellular regulated kinases (ERKs) has been reported to be a key factor interfering with the remodeling and proliferation of cartilage cells [29], and activation of the ERK pathway would further induce the synthesis of MMP-13. In addition, it has been reported that the interaction of ERK1-Smad1 protein could promote the development of OA [30].

**Figure 7:**
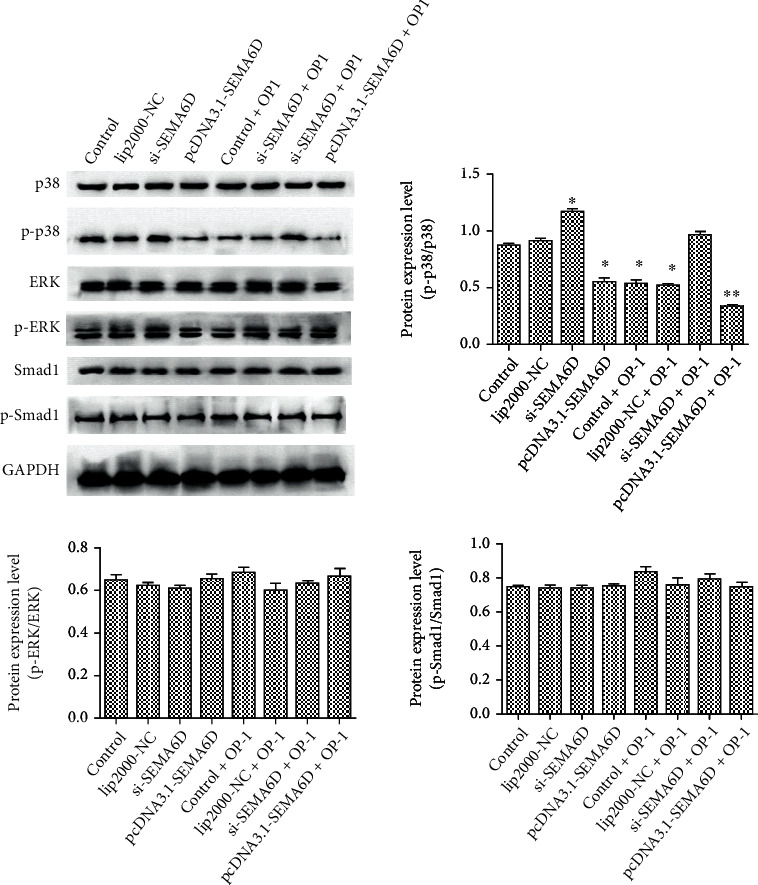
Evaluation of the regulatory pathway of SEMA6D. Results of western blotting showed that p38 presented responsive alteration when chondrocytes were treated with SEMA6D, whereas p-ERK and p-Smad1 did not show any change. ∗∗*p* < 0.01, ∗*p* < 0.05 compared with the control group.

For further investigation of the regulatory pathway of SEMA6D, si-SEMA6D and pcDNA3.1-SEMA6D were transfected into normal chondrocytes and OP1-treated C28/I2 chondrocytes. We then estimated the protein expressions of p38, ERK, and Smad1 via western blotting. As shown in Figure 5, the results of western blotting indicate that silencing SEMA6D using si-SEMA6D significantly improved the extent of phosphorylation of p38 in normal chondrocytes (Figure 7, *p* < 0.05), whereas up-regulation of SEMA6D decreased the phosphorylation level of p38 conspicuously in normal chondrocytes (*p* < 0.05) and OP1-treated C28/I2 chondrocytes (*p* < 0.001). However, no significant difference existed in the expression levels of ERK, p-ERK, Smad1, and p-Smad1, which might infer that SEMA6D mainly participated in regulating the p38 pathway.

**Figure 8:**
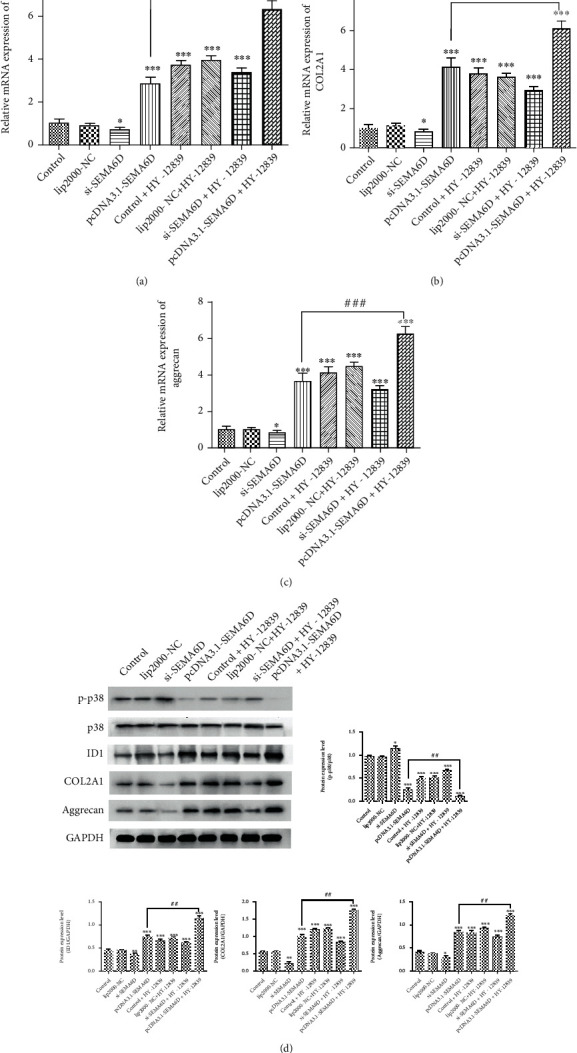
SEMA6D exerted regulatory effect in OA by inhibiting p38 pathway. The results of qRT-PCR of anabolism-related proteins including ID1 (a), COL2A1 (b), and Aggrecan (c). D. The expression levels of anabolism-related proteins including Aggrecan, ID1, and COL2A1 and p38 pathway-related proteins including p-p38 and p38 were measured by western blotting. ∗∗∗*p* < 0.001, ∗∗*p* < 0.01, ∗*p* < 0.05, compared with the control group; ^###^*p* < 0.001, ^##^*p* < 0.01.

For further evaluating the role of SEMA6D in regulating p38 pathway, we introduced p38 MAPK inhibitor HY-12839. First, C28/I2 chondrocytes were divided into two groups; of these, one group was treated with 30 nM of HY-12839 to block the signaling function of p38 pathway, and the other group was treated with the same volume of DMSO. Subsequently, si-SEMA6D, pcDNA3.1-SEMA6D, and lip-2000-NC were transfected into C28/I2 chondrocytes. Next, we scrutinized the expression level of anabolic process-associated proteins, including Aggrecan, collagen type II A1 (COL2A1), and ID1 proteins, which have been reported to be up-regulated in OA [31–33]. As shown in Figures 8(a)–8(c), up-regulation of SEMA6D using pcDNA3.1-SEMA6D could significantly induce the transcription of Aggrecan, COL2A1, and ID1 (*p* < 0.01) in C28/I2 chondrocytes without HY-12839 treatment, confirming that SEMA6D is a promotor of C28/I2 chondrocytes anabolism. In addition, in the HY-12839-treated group, the levels of Aggrecan, COL2A1, and ID1 were up-regulated compared with those in the control group (*p* < 0.01); overexpression of SEMA6D using pcDNA3.1-SEMA6D induced even higher levels of Aggrecan, COL2A1, and ID1 (*p* < 0.001). Moreover, the results of western blotting further verified the p38 suppressing effect of SEMA6D (Figure 8(d)). According to the results of western blotting, the expression level of phosphorylated p38 decreased on OP1 treatment (*p* < 0.01) and overexpression of SEMA6D, suggesting that there was lower activation of p38 in C28/I2 chondrocytes with active anabolism. The expressions of Aggrecan, COL2A1, and ID1 significantly increased upon up-regulation of SEMA6D in HY-12839-treated C28/I2 chondrocytes (*p* < 0.001). Based on these results, we can conclude that SEMA6D is validated in preventing the course of OA via inhibiting the activation of p38.

**Figure 9:**
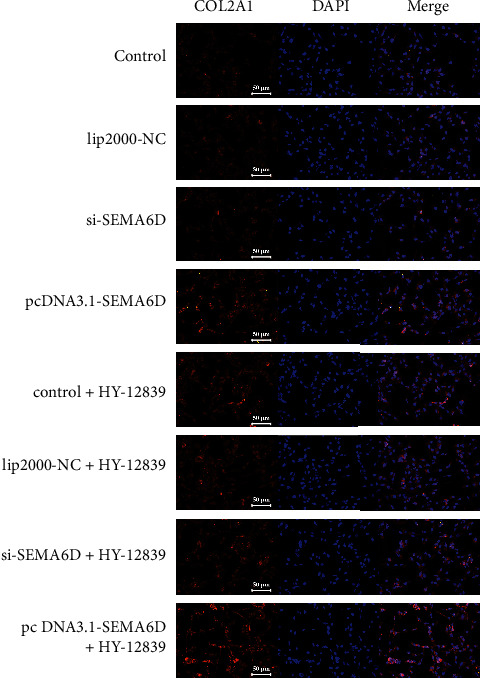
SEMA6D regulated the expression level of COL2A1 via p38 signaling pathway determined by immunofluorescence staining. Down-regulation of SEMA6D led to the lower fluorescence intensity that is the reduced expression of COL2A1, while up-regulation of SEMA6D displayed the opposite effect.

To confirm the effect of SEMA6D in improving OA, immunofluorescence staining was performed to further examine the expression of COL2A1. As shown in Figure 9, up-regulation of SEMA6D by pcDNA3.1-SEMA6D could increase the expression level of COL2A1 in normal chondrocytes. Furthermore, in C28/I2 chondrocytes with active anabolic metabolism induced by OP1, overexpression of SEMA6D visibly improved the levels of COL2A1. These results further verified that SEMA6D could improve the course of OA.

## 4. Discussion

OA is a common disease of the locomotor system characterized by the destruction of chondrocytes, and its incidence rate is positively correlated with age [34]. To date, the exact pathogenesis of OA remains unclear. Nevertheless, as research concerning the regulatory function of microRNAs in various types of diseases develops, the pathological association of miRNAs and OA has become a research hotspot, and accumulating studies have reported that miRNAs function as a key factor in mediating OA development. Wang et al. reported that microRNA-128a repressed chondrocyte autophagy and exacerbated knee OA by disrupting Atg12 [35]; microRNA-93 was considered to inhibit chondrocyte apoptosis and inflammation in OA by targeting the TLR4/NF-*κ*B signaling pathway [36]; miR-15a-5p is down-regulated in OA chondrocytes in relation to the up-regulated expression of PTHrP; and miR-15a-5p promotes the degeneration of chondrocytes by targeting PTHrP [37]. Simultaneously, our previous studies reported that miR-7 was significantly overexpressed in OA C28/I2 chondrocytes and served as a promoting factor in mediating the course of OA [12]. For further exploration of the downstream regulatory pathway, multiple databases including Targetscan, starBase v3.0, and miRanda were used to determine the target gene of miR-7, and SEMA6D was found to be a target gene with highest score. Bioinformatics analysis showed that there is an overlapping sequence between miR-7 and SEMA6D, indicating the possibility of interaction between miR-7 and SEMA6D. SEMA6D was then proved to be negatively associated with the miR-7. The results of immunohistochemical analysis showed that the expression level of SEMA6D was higher in normal human cartilage tissues than in OA tissues. A similar phenomenon could be found in rat models; that is, up-regulation of miR-7 resulted in lower expression level of SEMA6D, whereas blockage of miR-7 presented the opposite outcome.

Although SEMA6D has been found to be an abnormal factor in regulating the course of OA, whether SEMA6D mediates miR-7 levels in OA deserved further evaluation.

It is reported that the pathogenesis of OA comprises an imbalance of catabolic and anabolic activities of C28/I2 chondrocytes [17, 18]. Proper balance of anabolic and catabolic activities is crucial for the maintenance of cartilage tissue integrity and repair of molecular damages sustained during daily usage [17]. In individuals with degenerative diseases such as OA, this balance of anabolic and catabolic activities is compromised, and the extent of tissue degradation predominates over the capacity of tissue repair. In OA, an elevation of antianabolic and catabolic factors is observed. Anabolic activity is also increased, but this response fails to repair the tissue because of both quantitative and qualitative insufficiency [38]. Anabolic and catabolic activities involved in cartilage degeneration were responsible for tissue degeneration and critical to identifying and developing means to treat OA [39]. Curcumin supplementation was reported to enhance the production of bone marrow mesenchymal stem cells to promote the anabolism of articular chondrocytes and cartilage repair [40]. Further, Kdm6b has been reported to regulate cartilage development and homeostasis through anabolic metabolism [41].

Hence, we used the reported FN-f and OP1 to induce active catabolism and anabolism, respectively. The levels of miR-7 and SEMA6D were investigated, and we found that miR-7 was up-regulated in FN-f-treated C28/I2 chondrocytes, whereas the levels of SEMA6D decreased. The expression level of miR-7 and SEMA6D altered in a dose-dependent manner. In contrast, SEMA6D was overexpressed in OP1-treated C28/I2 chondrocytes, and miR-7 was down-regulated. Western blotting further confirmed this phenomenon. These results probably suggest that SEMA6D participated in regulating the catabolism and anabolism of C28/I2 chondrocytes.

Subsequently, si-SEMA6D and pcDNA3.1-SEMA6D were transfected into C28/I2 chondrocytes to further study the exact function of SEMA6D. The results showed that the up-regulation of SEMA6D led to lower quantities of MMP-2 and MMP-13 both in normal chondrocytes and in FN-f-induced OA chondrocytes, whereas the knockdown of SEMA6D had the opposite effect, indicating that SEMA6D can help prevent the development of OA. Next, we estimated the expression levels of factors involved in pathways pathologically correlated with OA to find the regulatory pathway of SEMA6D, and we found that p38 was a responsive target of SEMA6D. Hence, HY-12839, a p38 inhibitor, was added to C28/I2 chondrocytes for further exploration of the relationship between SEMA6D and p38 pathway. The results demonstrated that SEMA6D indeed exerted anabolism promoting effect via hindering the activation of the p38 pathway.

From the abovementioned studies, we can conclude that SEMA6D serves as a suppressor of OA and miR-7/SEMA6D axis could regulate the development of OA by mediating the p38 pathway. There are some limitations to this study. There was a lack of the application of SEMA6D in OA rat models *in vivo*. Further, we could not determine the time for which the observed effects lasted and the dose of SEMA6D. The in-depth studies for overcoming these limitations should be performed in the future.

## 5. Conclusions

The present research showed that SEMA6D is a negative regulatory factor of miR-7 and a pivotal mediator of catabolism and anabolism of C28/I2 chondrocytes. SEMA6D exerts its function by inhibiting the activation of the p38 pathway.

## Data Availability

The research data generated during the present study are available from the corresponding author on reasonable request.

## Abbreviations

OA: Osteoarthritis
SEMA6D: Semaphorin 6D
si-SEMA6D: Small interfering RNA of SEMA6D
FN-f: Fibronectin fragment
OP1: Osteogenic protein 1
MMP: Matrix metalloprotein
ERK: Extracellular regulated protein kinases
COL2A1: Collagen type II A1
p-p38: Phosphorylated p38.
